# Epigenetic mechanisms in preeclampsia: translational therapeutic strategies and precision-medicine perspectives

**DOI:** 10.25122/jml-2026-0058

**Published:** 2026-04

**Authors:** Oana-Eliza Crețu, Cristian Viorel Poalelungi, Adrian Valeriu Neacșu, Adina Nenciu, Iuliana Ceaușu

**Affiliations:** 1 Carol Davila University of Medicine and Pharmacy, Bucharest, Romania; 2 Dr. I. Cantacuzino Clinical Hospital, Bucharest, Romania

**Keywords:** preeclampsia, epigenetic therapy, DNA methylation, histone deacetylase, microRNA therapeutics, nanomedicine, precision obstetrics, AIP, artificial intelligence processing, cfDNA, cell-free DNA, C19MC, chromosome 19 microRNA cluster, CRISPR, clustered regularly interspaced short palindromic repeats, dCas9, deactivated CRISPR-associated protein 9, DNMT, DNA methyltransferase, DHA, docosahexaenoic acid, EPA, eicosapentaenoic acid, ENG, endoglin, EVs, extracellular vesicles, FLT1, fms-like tyrosine kinase 1, HAT, histone acetyltransferase, HDAC, histone deacetylase, HIF1A, hypoxia-inducible factor 1 alpha, HMT, histone methyltransferase, lncRNA, long non-coding RNA, miRNA, microRNA, NGS, next-generation sequencing, PE, preeclampsia, qPCR, quantitative polymerase chain reaction, RNA-seq, RNA sequencing, sEng, soluble endoglin, sFlt-1, soluble fms-like tyrosine kinase-1, SGA, small for gestational age, TSA, trichostatin A, VEGFA, vascular endothelial growth factor A

## Abstract

Preeclampsia is increasingly understood not only as a clinical syndrome of hypertension and organ dysfunction, but also as a disorder in which placental gene regulation, maternal vascular adaptation, and inflammatory signaling are shaped by abnormal epigenetic control. While several reviews have described epigenetic biomarkers in preeclampsia, the therapeutic implications of these mechanisms remain less clearly integrated. This review, therefore, focuses on the translational potential of epigenetic pathways as therapeutic entry points, with particular attention to DNA methylation, histone regulation, non-coding RNA networks, extracellular vesicle communication, and hypoxia-responsive placental signaling. Rather than treating these mechanisms solely as diagnostic signatures, the article evaluates how they may define molecular endotypes, identify pregnancies that could benefit from closer surveillance, and guide future interventions targeting upstream placental dysfunction. Potential strategies include selective modulation of DNMT and HDAC activity, microRNA inhibition or replacement, nutritional and environmental epigenetic optimization, placenta-oriented nanocarrier delivery, and pharmacogenomic stratification. The review also addresses the central barriers to translation, including tissue specificity, maternal-fetal safety, off-target epigenomic effects, ethical acceptability, long-term developmental consequences, and regulatory uncertainty. By reframing epigenetic alterations as actionable biological circuits rather than isolated biomarkers, this work provides a therapeutic and precision-medicine perspective on preeclampsia and outlines research priorities needed before epigenetic interventions can be responsibly evaluated in pregnancy.

## Introduction

Preeclampsia remains a major obstetric syndrome in which abnormal placentation, angiogenic imbalance, endothelial dysfunction, and maternal inflammatory activation converge after mid-gestation [1-4]. Although clinical management has improved, the available therapeutic armamentarium remains limited: surveillance, antihypertensive treatment, seizure prophylaxis when indicated, and delivery when maternal or fetal risk becomes unacceptable. This clinical reality creates a translational gap between the growing molecular understanding of the disease and the relatively narrow range of interventions currently available to modify its upstream biology.

Epigenetic regulation is particularly relevant to this gap because trophoblast differentiation, spiral artery remodeling, immune tolerance, and the response to hypoxia depend on tightly coordinated changes in DNA methylation, histone marks, and non-coding RNA expression [5-8]. In preeclampsia, these layers of regulation do not act as isolated abnormalities. They form interacting circuits that influence angiogenic signaling, oxidative stress, mitochondrial adaptation, extracellular vesicle communication, and maternal vascular reactivity. For this reason, epigenetic findings should be interpreted not only as disease markers but also as potential indicators of modifiable molecular vulnerability.

The present review is deliberately positioned as a translational and therapeutic synthesis. It examines how epigenetic mechanisms identified in preeclampsia could be converted into clinically meaningful strategies, including target discovery, molecular stratification, safer drug-delivery systems, and precision prevention. This focus distinguishes the article from reviews that focus primarily on diagnostic biomarkers or descriptive molecular alterations. The central question is not only which epigenetic signatures are altered in preeclampsia, but whether these signatures can inform interventions that are biologically plausible, placenta-aware, and acceptable for use in pregnancy.

Accordingly, the review addresses three objectives: first, to outline the epigenetic circuits that create a therapeutic rationale in preeclampsia; second, to evaluate candidate intervention classes such DNA methyltransferase (DNMT) and histone deacetylase (HDAC) modulation, microRNA-based approaches, nutritional epigenetic modifiers and nanotechnology-assisted delivery; and third, to define the safety, ethical and regulatory requirements that must be satisfied before such strategies can move from experimental models toward clinical research. The article therefore provides a framework for future precision obstetrics grounded in molecular endotyping and maternal-fetal safety.

### Epigenetic regulation in normal placental development

#### DNA methylation

DNA methylation plays a fundamental role in guiding normal placental development by regulating gene expression patterns required for trophoblast proliferation, differentiation, and the establishment of maternal-fetal immune tolerance [9]. The placenta displays a unique methylation landscape, characterized by extensive partially methylated domains and locus-specific methylation that differ markedly from those in somatic tissues [10]. These features support the highly dynamic transcriptional environment necessary for placental formation [11]. Proper methylation of genes involved in angiogenesis, cell invasion, and oxidative stress responses is essential for the coordinated development of the cytotrophoblast and syncytiotrophoblast layers [12]. Aberrant methylation can therefore disrupt trophoblast function, impair spiral artery remodeling, and alter placental vascularization, setting the stage for pregnancy complications. Understanding the baseline methylation architecture provides a critical reference against which disease-associated deviations can be evaluated.

#### Histone modifications

Histone modifications further refine the epigenetic control of placental gene expression by mediating chromatin accessibility and transcriptional activity [13]. Acetylation, methylation, phosphorylation, and ubiquitination of core histones occur in a tightly regulated and stage-specific manner during placental development, supporting the balance between trophoblast stemness and differentiation [14]. Key histone marks, such as H3K4me3 and H3K27ac, are associated with active promoters and enhancers that facilitate trophoblast invasion and endocrine function, whereas repressive marks, such as H3K27me3, contribute to lineage specification and selective gene silencing [15]. Enzymes, including histone acetyltransferases, deacetylases, methyltransferases, and demethylases, collaborate to maintain chromatin states compatible with the rapidly changing developmental demands of the placenta [16]. Disruption of these finely tuned processes can alter cellular identity, impair placental morphology, and predispose to pathological states [17].

#### Non-coding RNAs (miRNA, lncRNA, circRNA)

Non-coding RNAs represent a major layer of epigenetic regulation in the placenta, coordinating the transcriptional and post-transcriptional networks that underpin its development [18]. MicroRNAs are abundantly expressed in trophoblasts and play central roles in modulating cell invasion, angiogenesis, immune interactions, and adaptation to hypoxic conditions by targeting specific mRNAs [19]. Long non-coding RNAs shape gene expression by scaffolding chromatin-modifying complexes, regulating transcription factor activity, and influencing RNA stability [20]. Although less studied, circular RNAs contribute to regulatory crosstalk by acting as microRNA sponges or interacting with RNA-binding proteins, thereby modulating trophoblast behavior [21]. The placenta’s distinctive repertoire of non-coding RNAs reflects its dynamic and multifunctional nature, facilitating the tightly regulated processes essential for successful pregnancy establishment.

### Epigenetic crosstalk in trophoblast differentiation

Trophoblast differentiation relies on coordinated interactions between multiple epigenetic layers, creating a complex regulatory environment that integrates DNA methylation, histone modifications, and non-coding RNA activity [22]. Crosstalk between these mechanisms ensures that chromatin architecture, transcriptional programs, and post-transcriptional regulation operate seamlessly to support lineage commitment and functional maturation [23]. For instance, microRNAs can influence DNA methyltransferase expression, shaping methylation landscapes, while long non-coding RNAs recruit histone-modifying enzymes to specific genomic sites, reinforcing or antagonizing methylation-based regulation [24]. This interplay enables trophoblasts to rapidly adapt to environmental cues, including oxygen tension, immune signaling, and metabolic demands [25]. A comprehensive understanding of this integrated epigenetic framework is essential for interpreting how deviations in these processes may contribute to the development of pathological conditions.

### Epigenetic alterations in preeclampsia as therapeutic entry points

#### Angiogenic and hypoxia-responsive DNA methylation targets

In preeclampsia, altered DNA methylation affects genes involved in angiogenesis, hypoxia adaptation, immune signaling, and trophoblast differentiation [26-30]. From a therapeutic perspective, the relevance of these changes lies in their capacity to connect placental stress to downstream maternal disease. Hypomethylation or differential methylation involving fms-like tyrosine kinase 1 (FLT1), endoglin (ENG), hypoxia-inducible factor 1-alpha (HIF1A), and related hypoxia-responsive loci may favor anti-angiogenic signaling, endothelial injury, and inadequate vascular remodeling. These findings do not yet justify direct methylation editing in pregnancy, but they identify pathways in which selective, low-intensity modulation or upstream prevention could, in theory, restore a more favorable angiogenic balance.

#### Histone regulatory enzymes and chromatin plasticity

Histone modifications in preeclamptic placentas indicate altered chromatin accessibility at genes regulating trophoblast invasion, extracellular matrix remodeling, inflammation, and oxidative stress adaptation [31-36]. Reduced activating marks or enrichment of repressive marks may restrict trophoblast plasticity and impair the ability of placental cells to respond to changing oxygen tension. These observations provide a rationale for investigating histone-modifying enzymes as targets; however, broad HDAC or histone methyltransferase inhibition is unlikely to be acceptable in pregnancy without exquisite tissue specificity. The translational value of this field, therefore, resides in identifying safer isoform-specific or nutritionally mediated approaches rather than applying conventional oncology-derived epigenetic drugs.

#### Non-coding RNA networks as modifiable regulators

Dysregulated microRNAs and long non-coding RNAs contribute to several treatable biological domains in preeclampsia, including mitochondrial dysfunction, trophoblast apoptosis, immune activation, and endothelial maladaptation [37-42]. miR-210, miR-155, and miR-34a are repeatedly implicated in hypoxia, inflammation, and impaired cellular invasion, while selected lncRNAs influence chromatin architecture and vascular gene expression. Their therapeutic appeal derives from sequence-specific modulation through antagomiRs, mimics, or RNA sponges. The major unresolved challenge is delivery: any RNA-based intervention must reach the placenta or maternal vascular compartment with minimal fetal exposure and without broad immune activation.

#### Extracellular vesicles and placenta-directed delivery

Placental extracellular vesicles and exosomes are important not only as carriers of disease information, but also as models for future therapeutic delivery [43-45]. In preeclampsia, altered exosomal cargo can propagate placental stress to maternal endothelial and immune cells. The same biological principle could be used therapeutically if engineered vesicles or vesicle-inspired nanocarriers can deliver RNA molecules, small epigenetic modulators, or protective cargo to selected placental targets. This delivery-oriented interpretation makes extracellular vesicles central to the future therapeutic landscape rather than limiting them to biomarker discovery.

#### Hypoxia-epigenetic feedback loops

Hypoxia interacts bidirectionally with epigenetic regulation, stabilizing hypoxia-inducible factors and reshaping DNA methylation, histone marks, and non-coding RNA transcription [46-50]. These changes can reinforce anti-angiogenic signaling, oxidative stress, and inflammatory activation, creating a self-amplifying loop that contributes to disease progression. Therapeutic development should therefore prioritize interventions that can interrupt feedback loops rather than correcting isolated molecular abnormalities. Candidate approaches may include improving mitochondrial resilience, reducing oxidative stress, modulating hypoxia-induced microRNAs, or targeting downstream angiogenic imbalance.

## Methods

The literature search was designed to identify studies relevant to the translational use of epigenetic mechanisms in preeclampsia, with emphasis on therapeutic plausibility, target identification, biomarker-guided stratification, and safety considerations. Basic, translational, and clinical studies were considered when they examined DNA methylation, histone modifications, chromatin remodeling, non-coding RNAs, extracellular vesicles, liquid biopsy signatures, or epigenetically influenced angiogenic and inflammatory pathways. Studies addressing deoxyribonucleic acid methyltransferase (DNMT) or histone deacetylase (HDAC) modulation, microRNA-based strategies, nutritional epigenetic modifiers, nanotechnology-assisted delivery, or pharmacogenomic approaches were prioritized when they provided mechanistic or translational insight.

Articles were retrieved from PubMed and Web of Science using combinations of terms related to preeclampsia, placental epigenetics, epigenetic biomarkers, epigenetic therapy, DNA methylation, histone modification, microRNA, lncRNA, circRNA, chromatin remodeling, extracellular vesicles, nanomedicine, pharmacogenomics, and precision obstetrics (Table A1). Boolean operators were applied to capture both mechanistic studies and translational reports. Full-text, peer-reviewed English-language articles were selected to ensure that methodological details and molecular outcomes could be evaluated. The search strategy is summarized in Appendix A.

Appendix A

**Table 1:** Key limitations and factors influencing the diagnostic accuracy of epigenetic biomarkers in preeclampsia

Primary limitations	Additional contributing factors	References
Variability in sample sources (placenta, maternal blood, cell-free DNA, exosomes)	Differences in gestational age at sampling	[41,45]
Lack of validation in large or diverse cohorts	Maternal comorbidities (obesity, hypertension, diabetes)	[21,30]
Differences in analytical platforms (methylation arrays, sequencing, qPCR)	Environmental exposures (smoking, pollutants, nutrition)	[45,46]
Inter-laboratory variability and lack of methodological standardization	Technical variability in sample handling and storage	[8,14]
Limited reproducibility of single biomarkers across populations	Influence of medication use or pregnancy complications	[13,17]
Difficulty distinguishing mild from severe or early-onset from late-onset PE	Biological heterogeneity of placental pathology	[2,26]
Limited longitudinal data for predictive modeling	Ethnic and genetic background variation	[19,33]
Absence of clinically validated multi-marker epigenetic panels	Small sample sizes in many exploratory studies	[37,49]

**Figure 1:**
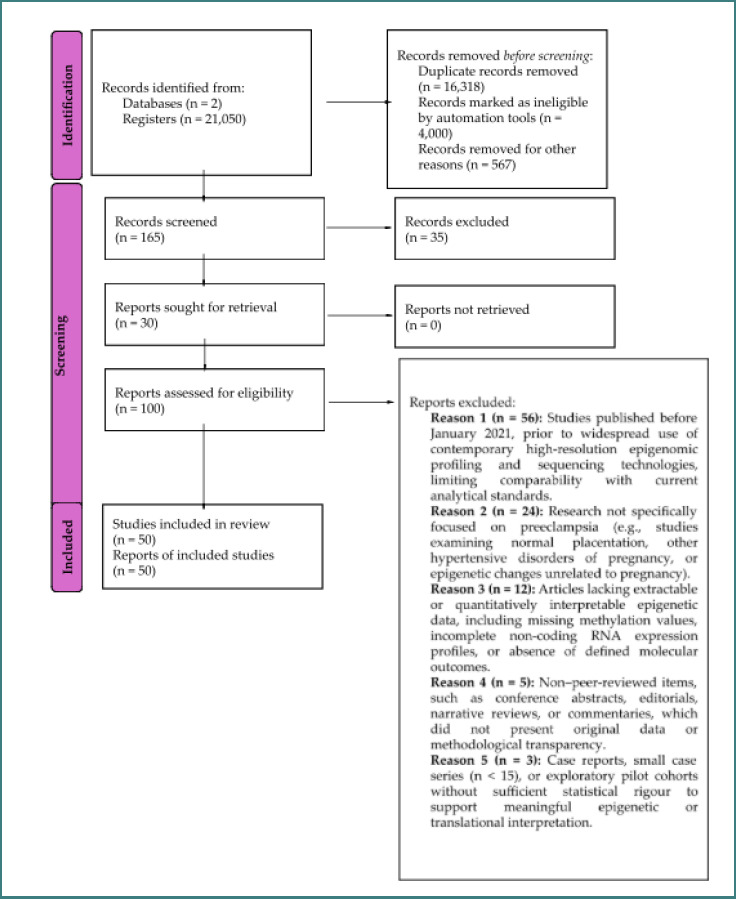
PRISMA flow diagram of articles related to epigenetic mechanisms in preeclampsia: therapeutic strategies and future perspectives

The PRISMA flow diagram was used to document the identification, screening, eligibility assessment, and final inclusion of studies (Figure 1). In interpreting the evidence, priority was given to studies that linked epigenetic alterations to clinically meaningful pathways such as trophoblast invasion, angiogenic imbalance, endothelial dysfunction, hypoxia signaling, oxidative stress, immune adaptation, or maternal-fetal outcomes.

After removal of duplicates and records considered ineligible before screening, the remaining articles were assessed by title and abstract, followed by full-text review when relevant. Studies were excluded if they did not focus on preeclampsia, lacked extractable epigenetic or translational data, were not peer-reviewed, or involved very small case-based evidence without sufficient methodological detail. The final synthesis retained studies that contributed to either the biological rationale for epigenetic targeting or the evaluation of future therapeutic and precision-medicine strategies.

Because the purpose of this review is translational rather than purely diagnostic, studies describing biomarkers were interpreted according to whether they helped define molecular endotypes, targetable pathways, or patient subgroups that might benefit from tailored surveillance or future intervention. Biomarker-only findings were therefore not treated as endpoints in themselves but as a step toward therapeutic stratification.

Two independent experts reviewed the eligible full-text articles and assessed study design, biological sample type, analytical technique, reported epigenetic outcomes, translational relevance, and limitations for maternal-fetal application. Particular attention was paid to the specificity of epigenetic modulation, potential fetal exposure, long-term developmental uncertainty, and feasibility of clinical implementation.

Disagreements were resolved through discussion until a consensus was reached. The synthesis is narrative and mechanistic, reflecting the heterogeneity of epigenetic platforms, biological samples, and outcome definitions. Consequently, the review aimed to map therapeutic opportunities and barriers rather than produce a quantitative meta-analysis of diagnostic performance.

Two independent experts in the field reviewed the full-text articles and examined the details of each study, including participant characteristics, methodological approaches, analytical techniques, and reported epigenetic outcomes. During this stage, studies were evaluated for scientific rigor, relevance to epigenetic mechanisms or therapeutic implications in preeclampsia, and the reliability of their sampling and data interpretation. Articles with inadequate methodological design, unclear analytical procedures, or outcomes that did not align with the aims of the review were excluded. Any discrepancies in judgment among the experts were resolved through discussion until a shared conclusion was reached, ensuring consistency and accuracy in selecting eligible basic, translational, and clinical studies.

## Results

### Epigenetic biomarkers as gateways to therapeutic stratification

Epigenetic biomarkers remain important, but in the context of this review, their principal value lies in defining biological subtypes that may eventually guide prevention, monitoring, or targeted intervention. DNA methylation profiles, circulating non-coding RNAs, and extracellular vesicle cargo can indicate whether a pregnancy is dominated by angiogenic imbalance, hypoxia signaling, inflammatory activation, or impaired trophoblast differentiation [4,8,9,34]. This interpretation shifts the focus from simple disease detection to therapeutic stratification.

Methylation changes involving angiogenesis and hypoxia-related genes may help identify placental phenotypes in which anti-angiogenic signaling is the dominant driver. Instead of being considered only as diagnostic markers, alterations in FLT1, ENG, HIF1A, and related pathways may support the selection of pregnancies for intensified surveillance, adjunctive vascular evaluation, or future targeted approaches aimed at restoring angiogenic equilibrium [12,18,25].

Non-coding RNA profiles may provide a second level of stratification by reflecting hypoxic, inflammatory, or apoptotic pathway activation. For example, miR-210, miR-155, and miR-34a are clinically relevant not only because they can be measured in maternal circulation, but because they regulate pathways that are theoretically modifiable through RNA-based interventions [23,24,37-41].

Extracellular vesicles add a delivery-oriented dimension to biomarker research. Their cargo mirrors placental stress, but their natural ability to transfer nucleic acids between cells also offers a conceptual model for future placenta-directed therapeutic systems [36,43-45].

Therefore, the most useful future epigenetic panels will likely be those that combine prediction with actionability. A clinically valuable panel should identify risk, suggest the dominant biological pathway, and help determine whether a patient is more likely to benefit from vascular, anti-inflammatory, nutritional, RNA-based, or intensified monitoring strategies.

### Circulating miRNAs as therapeutic signal maps

Circulating microRNAs are stable, measurable, and biologically informative, but their therapeutic importance extends beyond detectability [36,37,41]. Because they regulate trophoblast invasion, mitochondrial function, endothelial activation, and immune tolerance, they may act as signal maps of the dominant molecular stress affecting the placenta.

miR-210 is strongly linked to hypoxia and mitochondrial dysfunction, miR-155 to immune and endothelial activation, and miR-34a to apoptosis and impaired cellular proliferation [39-41]. These associations suggest possible intervention points, including antagomiR-based inhibition, restoration of protective microRNAs, or upstream modulation of hypoxia and oxidative stress. However, the pleiotropic nature of microRNAs means that therapeutic enthusiasm must be balanced by careful assessment of off-target effects.

The strongest translational scenario is not immediate clinical use of microRNA therapy, but the development of microRNA-informed endotypes. Such endotypes could support earlier recognition of severe biology, improve follow-up intensity, and provide a rational basis for future trials of placental-targeted RNA modulation.

Technical limitations remain important. Gestational age, sample processing, RNA extraction, normalization, maternal comorbidities, and medication exposure can influence circulating microRNA levels [42,44,45,50]. Standardization is therefore essential before these markers can be used to select patients for intervention.

When integrated with methylation markers, angiogenic factors, and clinical risk variables, microRNA profiles could become part of a multi-layered decision model for precision obstetrics rather than a stand-alone diagnostic test [28,29,50].

### DNA methylation markers as targets and safety signals

DNA methylation is attractive translationally because it is stable, measurable, and mechanistically linked to placental gene expression [32,35,42,45]. In preeclampsia, methylation patterns may identify both targetable pathways and potential safety signals, since any intervention that modifies methylation must avoid undesirable effects on fetal development.

Altered methylation involving fms-like tyrosine kinase 1 (FLT1), endoglin (ENG), hypoxia-inducible factor 1-alpha (HIF1A), endothelial PAS domain protein 1 (EPAS1), vascular endothelial growth factor A (VEGFA), and related pathways connects placental hypoxia to angiogenic imbalance and endothelial dysfunction [5,6,29,31,43]. These findings support a cautious therapeutic concept: rather than pursuing broad demethylation, future strategies should aim to stabilize pathogenic pathways through selective, local, or indirect modulation.

Placenta-derived cell-free DNA may help monitor molecular stress without invasive sampling [41,42,44,46]. In a therapeutic framework, such markers could be used to follow disease trajectory, evaluate response to preventive measures, or support trial enrichment for women with a specific molecular phenotype.

The main barrier is interpretability. Methylation patterns differ across placenta, whole blood, serum-derived cell-free DNA, and extracellular vesicles, while maternal body mass index, smoking, parity, and comorbidities may modify the epigenetic baseline [19-21,45].

Accordingly, methylation markers should be developed as components of integrated models that combine biological plausibility, clinical risk, and treatment-relevant stratification, rather than as isolated screening variables [10,13,15].

**Figure 2:**
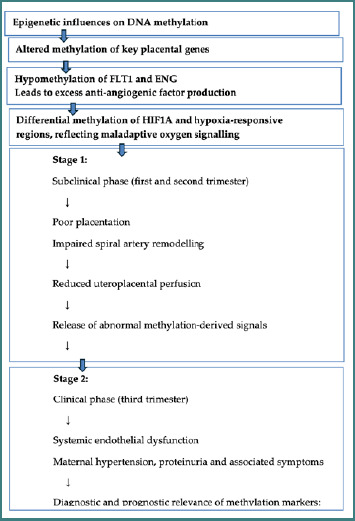
DNA methylation as a therapeutic entry point in preeclampsia

Figure 2 provides a conceptual overview of how methylation alterations can progress from early placental dysregulation to clinical disease, with potential intervention points. The sequence illustrates why methylation markers may be useful for both risk assessment and the identification of upstream biological circuits that could be targeted in future therapeutic research.

Figure 2 illustrates how early methylation disturbances may contribute to poor placentation, reduced uteroplacental perfusion, abnormal release of placental signals and later maternal endothelial dysfunction. In the context of this review, the figure should be interpreted as a translational pathway map: methylation signatures are relevant not only because they may support early diagnosis, but because they can identify angiogenic, hypoxic, and inflammatory circuits that could become targets for safer, more selective intervention.

### Liquid biopsy for monitoring molecular trajectory

Liquid biopsy approaches allow serial assessment of circulating cell-free DNA, RNA, and extracellular vesicles in maternal blood [46-49]. Their major therapeutic value is the possibility of following a molecular trajectory over time, rather than relying solely on a single diagnostic measurement.

Placenta-derived cell-free DNA can reflect trophoblast stress and altered methylation patterns involving loci such as RASSF1A, SERPINA3, TFPI2, or LINE-1 [41,45,46]. In future studies, these signatures may help identify when a pregnancy transitions from compensated placental dysfunction to clinically significant disease.

Extracellular vesicles provide complementary information because they carry microRNAs, long non-coding RNAs, circular RNAs, and DNA fragments protected from degradation [36,37,39,42]. Their cargo can indicate which regulatory networks are active and may also inform the design of biomimetic delivery systems.

A combined liquid-biopsy model could therefore serve three functions: early risk assessment, molecular endotyping, and longitudinal monitoring of response to preventive or investigational therapies. This approach is particularly relevant for distinguishing early-onset from late-onset disease, where the dominant molecular drivers may differ [17,48,49].

Before clinical translation, standardization of extracellular-vesicle isolation, sequencing platforms, reporting thresholds, and validation cohorts remains necessary [15,50].

### Diagnostic accuracy, actionability, and limitations

The diagnostic performance of individual epigenetic biomarkers remains variable because of differences in sample source, gestational timing, analytical platform, population characteristics, and disease definition [21,32,50]. For the purposes of therapeutic translation, the decisive question is not only whether a marker predicts preeclampsia, but whether it identifies a pathway that can be monitored, modified, or used to guide care. Multi-omic panels are therefore more likely to become clinically meaningful than isolated markers [33,34].

In clinical translation, epigenetic risk signatures should be interpreted alongside established obstetric surveillance tools. Previous Romanian conference data have described first-trimester ultrasound markers associated with preterm birth, supporting the broader value of early imaging-based risk assessment in pregnancies that require closer surveillance [51]. A related report focused specifically on ultrasound markers predictive of preeclampsia, reinforcing the need to combine molecular, clinical, and imaging parameters rather than interpreting epigenetic signatures in isolation [52].

Table 1 summarizes limitations that affect both diagnostic accuracy and therapeutic actionability of epigenetic biomarkers in preeclampsia.

Table 1 shows that methodological variability, biological heterogeneity, and confounding maternal factors limit the direct clinical use of epigenetic biomarkers. These barriers also affect therapeutic development: without robust sampling, reproducible platforms, and validated endotypes, it will be difficult to select appropriate patients, monitor treatment response, or define safety thresholds for future epigenetic interventions.

### Therapeutic strategies targeting epigenetic mechanisms

#### DNMT inhibitors and modulators

DNA methyltransferase (DNMT) inhibitors and modulators represent one of the most conceptually important but clinically delicate therapeutic directions in preeclampsia (Table 2) [29,32,35,43]. Because methylation influences angiogenic genes, trophoblast differentiation, and hypoxia signaling, controlled modulation could theoretically improve placental function. However, pregnancy requires a different therapeutic philosophy from oncology: broad demethylation is not acceptable, while fine-tuning of selected pathways may be more realistic.

**Table 2:** Translational epigenetic strategies in preeclampsia: therapeutic rationale, potential role, and pregnancy-specific limitations

Target/strategy	Mechanism	Potential role	Limitation	Safety issue
DNMT fine-tuning	Stabilizes methylation-sensitive angiogenic and hypoxia pathways	Adjunctive prevention or molecular-risk modulation	Global DNMT inhibition is unsafe	Fetal epigenome effects; long-term programming
HDAC / chromatin modulation	Restores chromatin accessibility for invasion and anti-inflammatory signaling	Experimental rescue of placental cell function	Most inhibitors are broad	Teratogenicity; off-target transcription
miRNA inhibition or replacement	Corrects hypoxia-, inflammation- or apoptosis-related networks	Future pathway-specific therapy or endotype-guided trials	Delivery and pleiotropic effects	Immune activation; fetal exposure
Nutritional epigenetic modifiers	Influence methyl donors, oxidative stress, and inflammatory tone	Low-risk adjunctive optimization in selected patients	Variable baseline status and dose-response	Over-supplementation; metabolic confounding
Placental nanodelivery	Targets RNA or epigenetic modulators to the maternal-placental interface	Platform for safer targeted interventions	Limited pregnancy biodistribution data	Placental transfer; fetal accumulation
Pharmacogenomic stratification	Integrates genetic and epigenetic variability in response	Precision surveillance and trial enrichment	Requires large, validated datasets	Equity, counseling, and interpretation issues

Source: elaboration by the authors. The table emphasizes that epigenetic intervention in pregnancy requires pathway specificity, placenta-aware delivery, and long-term developmental safety assessment.

Classical DNMT inhibitors such as 5-azacytidine and decitabine have mainly mechanistic value in this field, since their cytotoxicity and potential developmental risks preclude direct pregnancy use [5,42]. More plausible approaches include mild or indirect modulators of methylation, such as folate-related one-carbon metabolism, choline, betaine, and selected polyphenols, such as epigallocatechin gallate [22,25,28]. These interventions may influence methyl-donor availability, oxidative stress, and inflammatory tone without imposing global epigenomic disruption.

Experimental studies suggest that DNMT modulation can influence pathways involving FLT1, ENG, HIF1A, and placental angiogenic balance [29,33]. These data support further mechanistic work, but clinical development should prioritize dose, timing, target specificity, and long-term offspring follow-up.

A feasible near-term direction is not the use of strong epigenetic drugs in pregnancy, but the identification of low-risk interventions capable of stabilizing methylation-sensitive pathways before severe maternal disease develops [41-43].

#### HDAC inhibitors

Histone deacetylase (HDAC) inhibitors may restore chromatin accessibility in genes involved in trophoblast invasion, angiogenesis, immunoregulation, and resistance to oxidative stress [16-19,32]. In experimental models, HDAC inhibition can enhance trophoblast migration, regulate extracellular-matrix remodeling, and reduce inflammatory signaling [18,36].

Despite these promising mechanisms, the safety challenge is substantial. Most available HDAC inhibitors have broad activity, and broad chromatin modulation during pregnancy may produce unacceptable off-target or fetal effects [42,44]. Therefore, the therapeutic future of this class depends on isoform selectivity, placental targeting, short exposure windows, and rigorous developmental safety assessment.

Natural compounds with mild HDAC-modulatory or antioxidant effects, including resveratrol and curcumin, may offer a safer exploratory route, although bioavailability, dosing, and clinical efficacy remain unresolved [1,22].

#### miRNA-based therapeutics

MicroRNA-based therapeutics are attractive because they can, in theory, correct specific regulatory networks rather than globally modify the epigenome [24,37,49]. In preeclampsia, pathogenic upregulation of miR-210, miR-155, or miR-34a may be targeted with antagomiRs, locked nucleic acid inhibitors, or sponge strategies, whereas downregulated protective microRNAs may be restored with mimics.

Inhibition of miR-210 could improve mitochondrial function and trophoblast invasion; targeting miR-155 may reduce inflammation and endothelial activation; and modulation of miR-34a may protect against trophoblast apoptosis [21,39-41]. The relevance of miR-210 is further supported by recent systematic evidence describing its involvement in the pathogenesis and diagnostic assessment of preeclampsia, which justifies its inclusion among the most clinically relevant miRNA targets for future translational research [53]. These effects remain preclinical and require delivery systems that avoid widespread maternal or fetal exposure.

The most important translational requirement is placenta-oriented delivery. Lipid nanoparticles, polymeric carriers and engineered exosomes may improve stability, uptake and controlled release, but safety must be assessed across gestation and after birth [7,8,10,46,47].

Despite these advances, considerable challenges prevent immediate translation of miRNA-based therapies into clinical practice. Ensuring precise tissue targeting is essential to avoid off-target effects, given the pleiotropic actions of most microRNAs [2]. Stability in circulation, controlled release kinetics, and avoidance of immune activation remain technical hurdles [8]. Moreover, long-term epigenetic and developmental consequences for the fetus are unknown, necessitating rigorous safety evaluations in relevant pregnancy models [10]. Improvements in nanoparticle delivery systems, placenta-specific targeting ligands, and controlled dosing strategies may help mitigate these concerns [7].

#### Nutritional and environmental epigenetic modifiers

Nutritional and environmental modifiers are clinically appealing because they may influence epigenetic regulation while maintaining a more favorable safety profile than synthetic epigenetic drugs [12,16,45]. Their role should be understood as adjunctive and preventive rather than curative.

Vitamin D, methyl-donor nutrients, omega-3 fatty acids, and selected polyphenols can influence immune tolerance, oxidative stress, angiogenic balance, DNA methylation, and histone regulation [22,24,31,32,41,47,50]. These effects may be particularly relevant in women with nutritional deficiency, metabolic vulnerability, or inflammatory risk profiles.

However, nutritional epigenetic modulation should not be overstated. Clinical evidence remains heterogeneous, optimal dosing is uncertain, and the same intervention may have different effects according to baseline nutritional status, genotype, microbiome profile, and gestational timing [48,50].

Polyphenols, including epigallocatechin gallate (EGCG), resveratrol, and curcumin, possess antioxidant and anti-inflammatory properties that may counteract the oxidative stress characteristic of preeclampsia [12]. These molecules can inhibit DNMT and HDAC activity, thereby influencing both DNA methylation and histone acetylation [4]. In trophoblast and endothelial cell models, polyphenols have been shown to reduce oxidative damage, improve mitochondrial function, and modulate the production of angiogenic factors [36]. Although their bioavailability and metabolic stability are variable, polyphenols represent promising candidates for non-pharmacological epigenetic interventions [31].

Omega-3 fatty acids, particularly EPA and DHA, have been associated with improved vascular function, reduced inflammation, and enhanced placental perfusion [41]. These lipids may exert epigenetic effects by influencing the methylation of genes involved in lipid metabolism, endothelial signaling, and immune regulation [22]. Supplementation has been linked to altered microRNA expression profiles, including downregulation of pro-inflammatory microRNAs and modulation of angiogenesis-related pathways [24]. Omega-3 fatty acids may therefore contribute to stabilizing placental epigenetic patterns and offer protective effects against the development of preeclampsia, although evidence from clinical trials remains mixed [50].

Overall, nutritional and environmental epigenetic modifiers offer a complementary strategy to more targeted molecular therapies [3]. Their safety profile, accessibility, and potential to modulate multiple biological pathways make them attractive candidates for preventive or adjunctive interventions [49].

#### Nanotechnology-assisted epigenetic drug delivery

Nanotechnology-assisted delivery may be the key enabling technology for future epigenetic therapy in pregnancy [46-49]. The central obstacle is not merely identifying a molecular target but delivering the intervention to the relevant maternal-placental compartment while minimizing fetal exposure.

Lipid nanoparticles, polymeric nanoparticles, dendrimers, and exosome-mimicking vesicles can carry microRNA mimics, antagomiRs, small epigenetic modulators, or protective biological cargo [22,47,48]. Placental homing peptides, such as CGKRK or iRGD, offer a possible route toward tissue-enriched delivery [43].

Before clinical use, nanocarriers require careful evaluation of biodistribution, placental transfer, immune activation, persistence, fetal accumulation, and long-term developmental effects [10,12,14].

Another emerging approach involves the use of exosome-derived or exosome-like nanovesicles, which naturally possess high biocompatibility and intrinsic placental tropism [36]. These vesicles can be engineered to carry specific epigenetic cargo and may represent a physiologically harmonious mode of delivering therapeutic molecules with minimal immunogenicity [37].

Despite their potential, significant challenges must be addressed before nanotechnology-assisted epigenetic therapies can be translated into clinical practice. Safety remains a critical concern, as the pharmacokinetics, placental transfer patterns, and long-term developmental effects of nanomaterials require rigorous investigation [12]. Standardization of nanoparticle composition, size, surface chemistry, and dosing is essential to ensure reproducibility and minimize toxicity [14]. Moreover, ethical and regulatory considerations unique to pregnancy will influence the development and approval of such therapies [10].

#### Pharmacogenomics and personalized medicine

Pharmacogenomics and personalized medicine provide the framework through which epigenetic information may become clinically useful [32,35,40,41,43]. Preeclampsia is heterogeneous, and future interventions are unlikely to be equally effective across all phenotypes.

Genetic variants affecting one-carbon metabolism, angiogenic regulation, inflammatory signaling, microRNA binding, or drug metabolism may modify response to nutritional, RNA-based, or chromatin-directed interventions [4,11,23,24,45]. Similarly, epigenomic profiles could identify subgroups characterized by hypoxia-dominant, inflammatory-dominant, angiogenic-dominant, or metabolic-dominant disease.

A precision approach would integrate clinical risk, angiogenic biomarkers, epigenetic signatures, and pharmacogenomic variables to select monitoring intensity and, eventually, targeted interventions. This model requires large, diverse datasets and validated algorithms before it can be applied safely [29,47].

Advances in high-throughput sequencing, machine learning, and multi-omic data integration are expected to accelerate the development of personalized therapeutic algorithms [47]. These approaches can identify molecular subtypes of preeclampsia based on shared epigenetic features, thereby enabling the design of precision therapies tailored to specific pathological mechanisms rather than relying solely on clinical presentation [13]. However, several challenges remain, including the need for large, diverse datasets, standardized analytical pipelines, and comprehensive evaluation of long-term safety, particularly during pregnancy [29].

Despite these challenges, the convergence of pharmacogenomics and epigenetics represents a promising step toward a more individualized approach to managing preeclampsia. As the molecular landscape of the disorder becomes better understood, personalized medicine has the potential to enhance treatment efficacy, minimize adverse effects, and improve outcomes for both mother and fetus.

### Challenges, limitations, and ethical considerations

The development of epigenetic therapies for preeclampsia presents a range of scientific, clinical, and ethical challenges that must be addressed before such interventions can be safely translated into clinical practice [10,12]. Although targeting epigenetic pathways offers considerable promise, the complexity of these regulatory systems and their essential role in fetal development require careful and comprehensive evaluation [32].

A central scientific challenge relates to the specificity of epigenetic targets. Regulators such as DNMTs, HDACs, and microRNAs influence extensive gene networks rather than isolated pathways, which means that therapeutic modulation may lead to unintended off-target effects and altered gene expression patterns unrelated to preeclampsia [35,43]. Achieving precise modulation within placental tissues, without disturbing maternal or fetal systems, remains difficult [48]. New strategies, including the use of nanocarriers, placental homing peptides, and isoform-selective inhibitors, may increase target specificity, but these technologies are still in developmental phases and require substantial optimization [47,48].

Maternal–fetal safety is a major additional concern. Epigenetic interventions can induce stable shifts in DNA methylation or chromatin structure, raising questions about their potential influence on embryonic and fetal development [38]. Long-term and even transgenerational effects of epigenetic manipulation remain incompletely understood [25]. Moreover, physiological adaptations during pregnancy, such as altered drug metabolism, immune state, and vascular dynamics, affect pharmacokinetics and dosing accuracy, complicating the design of safe therapeutic regimens [50]. Preclinical models do not fully replicate human pregnancy, underscoring the need for phased, carefully controlled safety assessments [47].

Intervening in the fetal epigenome also raises notable ethical considerations. Epigenetic modifications during critical developmental periods may exert lifelong effects on metabolic health, neurodevelopment, and disease susceptibility [23]. Ethical concerns include the challenge of obtaining fully informed consent when long-term risks are uncertain, and the possibility that epigenetic therapies might inadvertently modify developmental trajectories [34]. These concerns underline the importance of rigorous ethical oversight and transparent communication when considering potential maternal–fetal interventions [5].

Regulatory hurdles add further complexity. Many epigenetic drugs, particularly DNMT and HDAC inhibitors, originate from oncology and possess known cytotoxic or teratogenic effects, making regulatory agencies cautious regarding their use in pregnancy [34]. Even safer or nutritionally derived epigenetic modulators must undergo extensive toxicological evaluation before approval for maternal–fetal application [45]. A lack of standardized protocols for assessing epigenetic drug safety in pregnancy further delays regulatory progress [50]. Agencies must also grapple with the novelty of epigenetic approaches, incomplete long-term data, and the difficulty of defining acceptable risk thresholds for both mother and child [32].

## Discussion

A major contribution of this review is the therapeutic reframing of epigenetic evidence. DNA methylation, histone modifications, non-coding RNAs, and extracellular vesicle cargo are not discussed solely as markers of disease presence but as indicators of biological circuits that might be monitored, stabilized, or targeted. This perspective is particularly important because current clinical treatment remains largely reactive and symptom-based.

The review supports a stepwise translational model. First, epigenetic signatures should be validated as reproducible indicators of molecular endotypes. Second, these endotypes should be linked to clinically meaningful outcomes such as early-onset disease, severe hypertension, fetal growth restriction, or maternal organ dysfunction. Third, only those pathways with biological plausibility and acceptable safety profiles should advance toward intervention studies.

Among candidate strategies, nutritional epigenetic optimization and improved risk stratification are closest to clinical feasibility, whereas DNMT/HDAC modulation, microRNA therapeutics, and CRISPR/dCas9 epigenetic editing remain experimental. Nanotechnology-assisted delivery may reduce off-target effects, but it also introduces new regulatory and safety questions specific to pregnancy [46-49].

This therapeutic orientation also changes how biomarkers should be evaluated. A marker that predicts disease but does not inform mechanism, timing, or intervention may be less clinically useful than a panel that defines a treatable pathway. Future biomarker development should therefore incorporate actionability, not only sensitivity and specificity.

The findings should be interpreted cautiously. Many available studies are small, heterogeneous, and platform-dependent, and most proposed epigenetic interventions remain preclinical. Cross-study comparison is limited by differences in biological samples, gestational timing, disease definitions, and analytical pipelines [45,46,48,50].

Future work should prioritize longitudinal cohorts, placental cell-type-specific profiling, standardized liquid-biopsy protocols, safety-focused pregnancy models, and integrated multi-omic datasets. Artificial intelligence may help identify molecular subtypes, but clinical deployment will require transparent algorithms and external validation.

The growing body of evidence on epigenetic alterations in preeclampsia has several practical implications for both clinical care and research. Epigenetic biomarkers derived from cell-free DNA, circulating microRNAs, and placental epigenomic signatures offer opportunities for earlier disease detection, enabling closer surveillance of high-risk pregnancies and the more timely implementation of preventive strategies. Integrating epigenetic indicators into existing risk models may improve diagnostic accuracy and allow for more personalized monitoring protocols. Moreover, understanding epigenetic dysregulation provides a mechanistic foundation for developing targeted therapies that address upstream placental dysfunction rather than merely treating clinical symptoms. These insights may also guide nutritional and lifestyle interventions during pregnancy, particularly for individuals with pre-existing metabolic or inflammatory vulnerabilities, by informing strategies to stabilize epigenetic patterns associated with placental health.

Despite significant progress, several limitations limit the immediate clinical translation of epigenetic discoveries. Many studies remain limited by small sample sizes, heterogeneous methodologies, and the absence of external validation in large, diverse populations. Differences in analytical platforms, normalization procedures, and sample types contribute to inconsistent findings and impede cross-study comparisons. Longitudinal data tracking epigenetic evolution across pregnancy are still scarce, limiting the ability to distinguish causal mechanisms from secondary responses. Ethical and safety concerns also constrain the development of epigenetic therapies, particularly those with the potential to influence fetal development or exert long-term epigenomic effects. Regulatory challenges and the lack of standardized guidelines for evaluating epigenetic interventions during pregnancy further delay translational progress.

Future research should focus on identifying early predictive epigenetic signatures that detect preeclampsia before clinical onset. Multi-marker panels integrating DNA methylation profiles, circulating microRNAs, and exosomal non-coding RNAs hold substantial promise for refining early screening and risk stratification. Combining epigenetic modulation with anti-angiogenic therapies may offer synergistic benefits by addressing both molecular and vascular components of the disease. Advances in genome engineering tools, including CRISPR/dCas9 epigenetic editors, open new avenues for precisely modifying disease-associated methylation or chromatin states, though their use in pregnancy will require stringent safety and ethical evaluation. Artificial intelligence and machine-learning approaches are expected to play an important role in analyzing large-scale epigenomic data, identifying molecular subtypes of preeclampsia, and supporting the development of personalized therapeutic algorithms. As technological capabilities continue to expand, integrating epigenomics with genetics, proteomics, metabolomics, and clinical phenotyping will be key to advancing precision medicine in preeclampsia.

## Conclusion

Preeclampsia is increasingly recognized as a disorder in which epigenetic dysregulation links early placental dysfunction to later maternal systemic disease. This review emphasized the translational and therapeutic implications of that biology, focusing on how DNA methylation, histone regulation, non-coding RNAs, and extracellular vesicle signaling may define actionable molecular circuits.

Epigenetic biomarkers remain valuable for early detection and risk stratification, but their greatest future impact may be in identifying molecular endotypes that guide surveillance, prevention, and targeted therapeutic development. This approach moves the field beyond descriptive biomarker discovery toward precision obstetrics.

Potential therapeutic strategies include selective DNMT or HDAC modulation, microRNA inhibition or replacement, nutritional epigenetic support, nanotechnology-assisted placental deliver,y and pharmacogenomic stratification. At present, these approaches should be considered investigational, with nutritional and monitoring-based applications being the most clinically plausible in the near term.

The principal barriers to implementation are target specificity, maternal-fetal safety, off-target epigenomic effects, long-term developmental uncertainty, ethical acceptability and regulatory approval. Future studies must therefore combine mechanistic innovation with rigorous safety evaluation and long-term follow-up.

Overall, epigenetic research in preeclampsia offers a pathway toward more precise, mechanism-based care. Its clinical promise will depend on transforming molecular signatures into validated, actionable, and safe strategies that improve outcomes for both mother and fetus
