# Supplementary material for: Epigenetic mechanisms in preeclampsia: translational therapeutic strategies and precision-medicine perspectives
Source: J Med Life. 2026 Apr;19(4):263–74. doi: 10.25122/jml-2026-0058 (PMC13252680; doi:10.25122/jml-2026-0058)
Supplement: Supplementary file 1 [file JMedLife-19-263-s001.pdf]

Appendix A

Table A1. Database search strategy

Database	Date of Last Search	Search Terms (MeSH + Keywords)	Boolean Logic / Query Structure	Filters Applied
PubMed	30 November 2025	preeclampsia, pre-eclampsia, hypertensive disorders of pregnancy, placental epigenetics, DNA methylation, histone modification, histone acetylation, chromatin remodeling, microRNA, miRNA, lncRNA, circRNA, non-coding RNA, epigenetic biomarker, cell-free DNA, trophoblast, placental hypoxia, angiogenesis	("preeclampsia"[MeSH] OR "pre-eclampsia" OR "hypertensive disorders of pregnancy") AND ("epigenomics" OR "epigenetics" OR "DNA methylation" OR "histone modification" OR "chromatin remodeling" OR microRNA OR lncRNA OR circRNA OR "non-coding RNA") AND ("placenta"[MeSH] OR trophoblast OR "placental development" OR "placental dysfunction" OR "cell-free DNA")	Humans; English; Publication date: 1 Jan 2021–30 Nov 2025
Web of Science	30 November 2025	preeclampsia, placental epigenetics, DNA methylation, histone acetylation, histone methylation, chromatin remodeling, microRNA, miRNA, lncRNA, circRNA, epigenetic regulation, exosomes, placental gene expression, cell-free DNA	TS = (preeclampsia OR "hypertensive pregnancy disorder") AND TS = ("DNA methylation" OR "histone modification" OR "chromatin remodeling" OR microRNA OR lncRNA OR circRNA OR "epigenetic marker" OR "epigenetic regulation") AND TS = (placenta OR trophoblast OR "placental development" OR "extracellular vesicles" OR "cell-free DNA")	Document type: Article; Language: English; Timespan: 2021–2025

© 2026 by the authors. This article is an open access article distributed under the terms and conditions of the Creative Commons Attribution (CC BY 4.0) license.
